# Are social inequalities in influenza vaccination coverage in Japan reduced by health policy?

**DOI:** 10.1016/j.pmedr.2019.100959

**Published:** 2019-07-25

**Authors:** Tselmuun Chinzorig, Kemmyo Sugiyama, Jun Aida, Toru Tsuboya, Ken Osaka

**Affiliations:** Tohoku University Graduate School of Dentistry, International and Community Oral Health Department, Japan

**Keywords:** Influenza, Vaccination, Inequalities, Awareness, Policy

## Abstract

Influenza vaccination is effective to prevent influenza infection. However, findings about association between socioeconomic status and influenza vaccination coverage are controversial. Online survey was conducted among 4995 participants between 20 and 69 years of age throughout Japan, January 2017. We asked about history of receiving vaccination in previous year and socioeconomic status, with their reasons for having vaccination or not. Age stratified multivariable logistic regression model was conducted to estimate the odds ratio (ORs) and 95% confidence intervals (CIs) of receiving vaccine for each educational level and income. Sex, self-reported health status, marital status and income were included as covariates. The rate for receiving influenza vaccine among ≤64 year-olds and ≥65 year-olds was 32.9% and 35.4%, respectively. Among younger adults, vaccination varied by each education: junior high school, 23.6%; senior high school, 27.2%; college, 32.6%; university, 36.2%; and graduate school, 39.8%. Compared to junior high school, those from graduate school tended to be more vaccinated (OR1.88, 95%CI 1.07–3.24). On the contrary, those aged above ≥65 years old received vaccination with no significant differences across education. Likewise, among respondents aged ≤64 year-olds, income was significantly associated with influenza vaccination. Despite being “Managed by school or company” (32.5%), having “No particular reason” was the frequent reason for both receiving influenza vaccination (23.8%) or not (34.3%). Adults with higher educational level were significantly more likely to receive vaccination. Subsidizing influenza vaccination may reduce inequality in receiving vaccination for adults. Strengthening vaccination through various approaches is necessary, such as managing by school or company.

## 1 Introduction

Influenza is associated with substantial morbidity and mortality. The global annual influenza-associated respiratory death is estimated to be 291,243 to 645,832 (Iuliano et al., 2018). Increasing influenza vaccination coverage is mandatory in public health sector. Although previous studies demonstrate social inequalities on pandemic mortality (Tricco et al., 2012; Mamelund, 2006; Grantz et al., 2016; Bengtsson et al., 2018; Rutter et al., 2012; Kee et al., 2007; Böhmer et al., 2012; Endrich et al., 2009; Chiatti et al., 2010; Vaux et al., 2011; Damiani et al., 2007; Ryu et al., 2011), further studies are necessary to discover social inequalities in influenza vaccination coverage risk factors. Added to this, in order to aid the international goals of reducing social inequality in health and ensuring good health for all by 2030 (*Reduce Inequality Within and Among Countries*, n.d.), reducing social inequality in health is a core aim. However, latest review of international and national pandemic preparedness plans demonstrates that this perception missing in this policy area (Mamelund, 2017).

During pandemics, particularly young children, older population and in general people who are already ill are vulnerable. Yet, younger adults are effected the most (Shanks and Brundage, 2012; Simonsen et al., 2013). According to the evidence that annual influenza vaccination is effective and safe with potential benefit in all age group, an expansion of the target populations includes adults aged 19 to 49 years old (Fiore et al., 2010). In 2010, the US Centers for Disease Control and Prevention (CDC) has recommended annual influenza vaccination for all individuals aged ≥6 months without contraindications (Centers for Disease Control and Prevention, n.d.).

Influenza vaccination is an important matter for Japan due to rapidly increasing aging population compare to other countries (Muramatsu and Akiyama, 2011). In Japan, according to the Preventive Vaccination Law the target group for annual vaccination are those aged ≥65 years old and those with certain chronic medical conditions, aged ≥60 (Enami and Otsubo, n.d.). This law enables local governments to partially subsidize influenza vaccination cost. Therefore, there is a possibility that trends of socioeconomic inequalities in older and younger adults are different in Japan.

This study aimed to investigate the association between individual's educational attainment, income as proxies for socioeconomic status (SES) and influenza vaccine reception. In addition, this study searched whether the association of SES differed by age because of the Preventive Vaccination Law in Japan and searched underlying reasons that influenced individuals' behavior of receiving vaccination or not.

## 2 Materials and methods

### 2.1 Settings and participants

This was a web-based cross-sectional study in Japan. The survey with self-administered questionnaire was conducted by Macromill Inc. in January 2017. This global research company has access to approximately 3 million registered individuals. They are able to specify the surveyed population by age and sex. According to our target population, 20–69 year olds, the company have distributed the survey through all prefectures of Japan. Those who agreed to answer the questionnaire participated in the survey to receive point-based incentives that can be converted into cash afterwards. Our survey ended until even number of responses for each sex and age-groups were acquired. Five participants had missing value for educational attainment, thus the study included 4995 respondents.

### 2.2 Dependent variable

The uptake of influenza vaccine was dependent variable and data on receiving vaccination was collected through following questions: ‘*Have you received influenza vaccination in the past year?*’, ‘*Received vaccination*’ and ‘*Did not receive*’ as response choices.

### 2.3 Independent variables

As independent variables, educational attainment and income were used as proxy measures of socioeconomic status. Educational attainment was divided in five categories: ‘Junior high school’, ‘Senior high school’, ‘Vocational and Technical College’, ‘University’ and ‘Graduate school’. Annual household income was categorized into four: lowest (<3 million JPY); 2nd (3–4.9 million JPY); 3rd (5–7.9 million JPY); top quartile (≥8 million JPY).

### 2.4 Covariates

Sex, self-reported current health status, and marital status were adjusted as covariates. Marital status was categorized as married, divorced or lost and single.

### 2.5 Reasons toward receiving influenza vaccination

To examine individuals' behavioral reason toward influenza vaccination, multiple choice question was prepared, some of which were used in previous studies (Iwasa and Wada, 2013). Participants who did not receive influenza vaccine were asked about inhibiting factors: *1) Economic reason*, *2) Scariness toward injection*, *3) No time to visit hospital*, *4) Fear of side effects*, *5) No experience of influenza*, *6) Feelings of vaccine to have no effectiveness*, *7) No necessity because children had grown up already*, *8) No elders living with*, *9) No particular reason*, *10) Others*. On the other hand, those who received the influenza vaccine were asked about motivating factors and stimulus: *1) Managed by school or company*, *2) Advice from medical person*, *3) Advice from family member*, *4) Feelings of vaccine being effective*, *5) Experience of severe influenza*, *6) Living with children*, *7) Living with elders*, *8) Living with a student at present preparing for entrance exam*, *9) No particular reason*, and *10) Others*.

In order to analyze whether the reasons toward influenza vaccination differed between educational attainments, we combined participants according to the years of educational attainment.

### 2.6 Statistical analysis

To demonstrate the association between influenza vaccine coverage and SES variables, multivariate logistic regression was done with an adjustment of sex, self-reported current health, and marital status as covariates. The main analysis was stratified by age groups, 20–64 years and ≥65 years based on two reasons. First, according to WHO guideline, 65 years and over are recommended to take influenza vaccine (WHO, 2018). Second, based on the current national immunization program in Japan, elders and high-risk individuals can receive subsidies by the municipalities. Chi-square goodness-of-fit test was 0.41 for 20–64-year-old regression model and 0.10 for ≥65-year-old model. Multivariate logistic regression analysis was performed to calculate adjusted odds ratios (aORs) and 95% confidence intervals (95% CIs). Pearson chi-square was applied to compare the reasons by educational attainment. We used Stata MP version 15.0 (Stata Corp., College Station, TX, USA) for statistical analysis. All tests were two-sided and statistical significance was considered as p < 0.05.

### 2.7 Ethical considerations

We considered each participant's response to the questionnaire as their consent to participate in the survey. The study protocol was reviewed and approved by the Ethics Committee of Tohoku University Graduate School of Dentistry.

## 3 Results

A total of 4995 participants participated in the survey. Numbers of males and females were approximately equally recruited, despite the number of respondents aged 65 years old or above were relatively small compared to participants of 20–64 years old. With respect to influenza vaccination, 32.9% of younger adults and 35.4% of elders had received influenza vaccination (Table 1). A slightly higher proportion of participants who lost their partner, divorced or single responded as not having received vaccination.

**Table 1:** Demographic characteristics and influenza vaccination.

Characteristics	Influenza vaccination (n = 4995)			
	20–64 years old (n = 4521)		≥65 years old (n = 474)	
	Did not receive	Did receive	Did not receive	Did receive
N (%)	3032 (67.1%)	1489 (32.9%)	306 (64.6%)	168 (35.4%)

Sex				
Male	1519 (67.4%)	733 (32.6%)	158 (64.5%)	87 (35.5%)
Female	1513 (66.7%)	756 (33.3%)	148 (64.6%)	81 (35.4%)

Age				
20–29 years old	651 (65.2%)	348 (34.8%)	–	–
30–39 years old	661 (66.2%)	338 (33.8%)	–	–
40–49 years old	661 (66.2%)	337 (33.8%)	–	–
50–59 years old	682 (68.3%)	317 (31.7%)	–	–
60–64 years old	377 (71.7%)	149 (28.3%)	–	–
65–69 years old	–	–	306 (64.6%)	168 (35.4%)

Marital status				
Married	1539 (63.8%)	874 (36.2%)	225 (61.3%)	142 (38.7%)
Divorced/lost	216 (73.0%)	80 (27.0%)	53 (74.6%)	26 (25.3%)
Single	1277 (70.5%)	535 (29.5%)	28 (77.8%)	8 (22.2%)

Current health				
Good	2246 (67.2%)	1094 (32.7%)	252 (66.1%)	129 (33.9%)
Poor	786 (66.5%)	395 (33.5%)	54 (58.1%)	39 (41.9%)

Educational attainment				
Junior high school	68 (76.4%)	21 (23.6%)	12 (85.7%)	2 (14.3%)
Senior high school	864 (72.8%)	323 (27.2%)	121 (65.4%)	64 (34.6%)
Vocational and technical college	731 (67.4%)	353 (32.6%)	56 (66.7%)	28 (33.3%)
University	1195 (63.8%)	677 (36.2%)	106 (60.9%)	68 (39.1%)
Graduate school	174 (60.2%)	115 (39.8%)	11 (64.7%)	6 (35.3%)

Annual household income (JPY)				
<3 million	829 (72.7%)	312 (27.3%)	102 (67.6%)	49 (32.4%)
3–4.9 million	832 (69.5%)	365 (30.5%)	105 (65.2%)	56 (34.8%)
5–7.9 million	789 (65.1%)	424 (34.9%)	68 (64.1%)	38 (35.9%)
≥8 million	582 (60.0%)	388 (40.0%)	31 (55.4%)	25 (44.6%)

Among 20–64 years old participants 1489 (32.9%) were vaccinated and the influenza vaccine coverage rate increased according to levels of educational attainment: junior high school, 23.6%; senior high school, 27.2%; vocational and technical college, 32.6%; university, 36.2%; and graduate school, 39.8%. Also, influenza vaccination coverage increased by each income level: <3million, 27.3%; 3–4.9 million, 30.5%; 5–7.9 million, 34.9% and >8 million, 40.0%.

Results from univariate analysis among ≤64-year-old shows that influenza vaccine coverage was significantly associated with SES. Especially, for participants who graduated from graduate school, the odds-ratio was OR 2.14, 95% CI 1.24 to 3.68 compared with individuals graduated from junior high school (Table 2). After adjusting age, sex, current health, marital status and annual household income, those who graduated from graduate school were at higher odds of influenza vaccination (OR 1.87, 95% CI 1.07 to 3.24, *p* value = 0.027) compared with junior high school. On the other hand, no significant associations were observed among participants of age ≥ 65 years.

**Table 2:** Odds ratio (95% confidence interval) on receiving influenza vaccination calculated by age stratified logistic regression.

		20–64 years old					≥65 years old					
	Crude OR	95% CI	*P-*value	aOR	95%CI	*P-*value	Crude OR	95%CI	*P-*value	aOR	95%CI	*P-*value
Education attainment												
Junior high school	Ref			Ref			Ref			Ref		
Senior high school	1.21	[0.73–2.01]	0.459	1.09	[0.66–1.82]	0.737	3.17	[0.69–14.62]	0.138	2.92	[0.62–13.75]	0.175
Vocational and technical college	1.56	[0.94–2.59]	0.083	1.34	[0.80–2.23]	0.261	3	[0.63–14.34]	0.169	2.81	[0.57–13.74]	0.203
University	1.83	[1.12–3.02]	0.017	1.56	[0.94–2.59]	0.083	3.85	[0.84–17.73	0.084	3.51	[0.75–16.51]	0.112
Graduate school	2.14	[1.24–3.68]	0.006	1.87	[1.07–3.24]	0.027	3.27	[0.54–19.75]	0.196	2.90	[0.47–18.14]	0.254

Sex												
Male	Ref			Ref			Ref			Ref		
Female	1.03	[0.91–1.17]	0.582	1.10	[0.96–1.25]	0.167	0.99	[0.68–1.45]	0.975	1.18	[0.77–1.82]	0.451

Marital status												
Married	Ref			Ref			Ref			Ref		
Divorced/lost	0.65	[0.50–0.85]	0.002	0.74	[0.56–0.97]	0.032	0.54	[0.30–0.95]	0.035	0.55	[0.30–1.01]	0.054
Single	0.74	[0.65–0.84]	<0.001	0.80	[0.69–0.92]	0.002	0.45	[0.20–1.02]	0.056	0.49	[0.21–1.14]	0.097

Current health status												
Good	Ref			Ref			Ref			Ref		
Poor	1.03	[0.90–1.19]	0.664	0.88	[0.77–1.02]	0.094	1.41	[0.89–2.24]	0.145	0.67	[0.42–1.09]	0.108

Annual household income (JPY)												
<3 million	Ref			Ref			Ref			Ref		
3–4.9 million	1.29	[0.94–1.77]	0.115	1.10	[0.92–1.32]	0.300	0.84	[0.20–3.59]	0.816	0.94	[0.57–1.56]	0.820
5–7.9 million	1.76	[1.28–2.42]	<0.001	1.26	[1.04–1.51]	0.017	1.1	[0.25–4.79]	0.903	1.05	[0.60–1.83]	0.965
≥8 million	2.05	[1.45–2.91]	<0.001	1.47	[1.20–1.79]	<0.001	0.94	[0.19–4.59]	0.941	0.79	[0.16–4.01]	0.775

aOR-Adjusted for sex, current health status, marital status and annual household income.

Likewise, among ≤64 year-old respondents' annual household income was significantly associated with influenza vaccination. Particularly, participants with >8million annual household income, the odds-ratio was OR 2.05, 95% CI 1.45 to 2.91 compared to lowest quantile. After adjusting the covariates, respondents with annual household income from top quantile were at significantly higher odds of influenza vaccination, compared to those with lowest quantile (OR 1.47, 95%CI 1.20 to 1.79, *P* ≤ 0.001). There was no statistical significance between annual household income and influenza vaccination among participants of age ≥ 65 years.

Table 3, the major reason among participants who did not receive the vaccine was “*No particular reason*” (34.3%), followed by “*Feelings of vaccine to have no effectiveness*” (19.3%), “*No experience of influenza*” (19.3%), “*Economic reason*” (15.2%), “*No time to visit hospital*” (13.1%), “*Scariness toward injection*” (10.7%), “*Fear of side effects*” (10.7%), “*Others*” (3.4%), “*No necessity because children had grown up already*” (2.1%) and “*No elders living with*” (1.7%). Among younger adults, when we stratified by educational attainment on the reasons of not having received influenza vaccine, “No experience of influenza” (P = 0.049), “Economic reason” (P = 0.001) and “No time to visit hospital” (P < 0.001) had statistical difference between educational attainment.

**Table 3:** Reasons for not receiving influenza vaccination stratified by age.

	Total	20–64 years old (n = 3032)				≥65 years old (n = 306)			
Reasons	(n = 3338) n %	Junior and senior high school, n = 932 (30.7%)	Vocational and technical college, n = 731 (24.1%)	University and graduate school, n = 1369 (45.2%)	*p* value	Junior and senior high school, n = 133 (43.5%)	Vocational and technical college, n = 56 (18.3%)	University and graduate school, n = 117 (38.2%)	*p* value
No particular reason	1144 (34.3)	331 (35.5)	241 (33.0)	476 (34.8)	0.543	38 (28.6)	23 (41.1)	35 (29.9)	0.218
Feelings of vaccine to have no effectiveness	645 (19.3)	154 (16.5)	131 (17.9)	277 (20.2)	0.071	35 (26.3)	10 (17.8)	38 (32.5)	0.124
No experience of influenza	644 (19.3)	170 (18.2)	151 (20.7)	224 (16.4)	0.049	40 (30.1)	15 (26.8)	44 (37.6)	0.275
Economic reason	508 (15.2)	183 (19.6)	119 (16.3)	186 (13.6)	0.001	14 (10.5)	1 (1.8)	5 (4.3)	0.038
No time to visit hospital	436 (13.1)	120 (12.9)	73 (10.0)	234 (17.1)	<0.001	4 (3.0)	1 (1.8)	4 (3.4)	0.836
Scariness toward injection	357 (10.7)	95 (10.2)	72 (9.8)	162 (11.8)	0.281	8 (6.0)	7 (12.5)	13 (11.1)	0.238
Fear of side effects	357 (10.7)	78 (8.4)	84 (11.5)	141 (10.3)	0.095	23 (17.3)	7 (12.5)	24 (20.5)	0.429
Others	113 (3.4)	32 (3.4)	32 (4.4)	37 (2.7)	0.123	6 (4.5)	4 (7.1)	2 (1.7)	0.203
No necessity because children had grown up already	69 (2.1)	24 (2.6)	20 (2.7)	21 (1.5)	0.107	1 (0.7)	–	3 (2.6)	0.288
No elders living with	58 (1.7)	12 (1.3)	16 (2.2)	24 (1.7)	0.369	2 (1.5)	1 (1.8)	3 (2.6)	0.829

In contrast, in Table 4, those who did receive the influenza vaccine, the most chosen reason was “*Managed by school or company*” (32.5%), followed by: “*No particular reason*” (23.8%), “*Advice from family member*” (15.4%), “*Feelings of vaccine being effective*” (13.4%), “*Living with children*” (8.9%), “*Advice from medical person*” (8.0%), “*Experience of severe influenza*” (7.4%), “*Living with elders*” (4.8%), “*Others*” (3.5%) and “*Living with a student at present preparing for entrance exam*” (2.2%). Stratification on the reason for receiving influenza vaccine showed that among younger adults, “Managed by school or company” (P = 0.006), “No particular reason” (P = 0.008) and “Advice from family member” (P = 0.002) had statistical difference among educational attainment.

**Table 4:** Reasons for receiving influenza vaccine stratified by age.

	Total	20–64 years old (n = 1489)				≥65 years old (n = 168)			
Reasons	(n = 1657) n %	Junior and senior high school, n = 344 (23.1%)	Vocational and technical college, n = 353 (23.7%)	University and graduate school, n = 792 (53.2%)	*p* value	Junior and senior high school, n = 66 (39.3%)	Vocational and technical college, n = 28 (16.7%)	University and graduate school, n = 74 (44%)	*p* value
Managed by school or company	538 (32.5)	104 (30.2)	116 (32.9)	311 (39.3)	0.006	3 (4.5)	1 (3.6)	3 (4.1)	0.975
No particular reason	395 (23.8)	99 (28.8)	87 (24.6)	162 (20.4)	0.008	20 (30.3)	10 (35.7)	17 (23.0)	0.381
Advice from family member	255 (15.4)	49 (14.2)	34 (9.6)	141 (17.8)	0.002	11 (16.7)	3 (10.7)	17 (23.0)	0.323
Feelings of vaccine being effective	222 (13.4)	33 (9.6)	46 (13.0)	102 (12.9)	0.252	13 (19.7)	8 (28.6)	20 (27.0)	0.514
Living with children	148 (8.9)	37 (10.8)	30 (8.5)	74 (9.3)	0.587	5 (7.6)	–	2 (2.7)	0.171
Advice from medical person	133 (8.0)	27 (7.8)	30 (8.5)	52 (6.6)	0.466	10 (15.1)	2 (7.1)	12 (16.2)	0.489
Experience of severe influenza	122 (7.4)	29 (8.4)	24 (6.8)	57 (7.2)	0.681	4 (6.1)	3 (10.7)	5 (6.8)	0.715
Living with elders	80 (4.8)	16 (4.6)	13 (3.7)	26 (3.3)	0.532	12 (18.2)	4 (14.3)	9 (12.2)	0.604
Others	58 (3.5)	7 (2.0)	16 (4.5)	23 (2.9)	0.148	6 (9.1)	2 (7.1)	4 (5.4)	0.700
Living with a student at present preparing for entrance exam	37 (2.2)	7 (2.0)	10 (2.8)	19 (2.4)	0.789	–	–	1 (1.3)	0.528

## 4 Discussion

This study showed that 20–64 year-old participants with higher educational attainment and higher annual household income had significantly higher reception of influenza vaccination compared to those with lower educational attainment and lower annual income. On the contrary, no socioeconomic inequalities were observed among those ≥65 years who were covered by the Preventive Vaccination Law in Japan. Therefore, subsidies for receiving influenza vaccination by the municipalities had the potential to reduce social inequalities in vaccination coverage. To the best of our knowledge, previous studies only investigated for inequalities on pandemic mortality (Mamelund, 2006; Grantz et al., 2016; Bengtsson et al., 2018; Rutter et al., 2012) and rarely on vaccination coverage. “No particular reason” was the most frequent response among participants who had not received vaccination. On the contrary, those being “Managed by school or company” had higher vaccine uptake among participants receiving influenza vaccine. Perceived reasons that had statistical significance according to educational attainment were “Managed by school or company” followed by “Advice from family member” and “No particular reason”, among younger adults. When implementing policy for improvement in vaccine coverage among younger adults, management through school or company should be considered besides subsidy may have no effect among younger and older adults.

In Japan, nationwide influenza vaccination program with subsidy was launched for people only aged ≥65 since 2001/2002 season (Fiore et al., 2010; Centers for Disease Control and Prevention, n.d.). However, in our study, the influenza vaccination coverage was almost equal between adults aged ≤64 years old and those aged ≥65 years old. Thus, according to our results, the nationwide influenza vaccination program with subsidy for people aged ≥65 did not seem to play an active role in increasing the influenza vaccination coverage in older adults. Studies on vaccination rate between younger and older adults had been rare due to the subsidies service in Japan to be unique from other developed countries.

Based on our result, older adults have lower influenza vaccination and this value is much lower than WHO global targets for vaccination coverage rates. It may be due to many municipalities provide subsidies only partially and does not have a recommended coverage goal. On the contrary, in the US, Medicare Part B covers the entire cost of the influenza vaccination for elders (https://www.medicare.gov/what-medicare-covers/part-b/what-medicare-part-b-covers.html, n.d.), and the Healthy People 2020 targets at least 90% coverage for adults aged ≥65 years (Healthy People 2020, n.d.). Therefore, these methods, we assume can be organized by municipalities in Japan.

“*No particular reason*” was the most frequent response among participants who had not received vaccination. On the contrary, those being “*Managed by school or company*” had higher vaccine uptake among participants receiving influenza vaccine. Among those being 20–64 years of age, “*Managed by school or company*” significantly differed between the educational attainment followed by “*No particular reason*” and “*Advice from family member*”. These points should be considered in vaccination reception because influenza vaccination coverage in Japan is still low.

Among those who received influenza vaccination, mostly managed by their company or school and received the vaccine for no particular reason was observed. By contrast, the participants who did not receive influenza vaccine had no particular reason on first of all reasons. The difference between previous studies and our results may elucidate that public concerns about influenza vaccination changed over time (Iwasa and Wada, 2013; Nawa et al., 2016; Hothersall et al., 2012). Among 20–64 year olds, having no experience of influenza, having economic reason and lacking time to visit hospital significantly differed between educational attainments. Strengthening any organization or institutions management and policy to recommend individuals, especially younger adults, to receive influenza vaccine could be one of the effective tools to increase influenza vaccine coverage.

Moreover, there was a difference in the reasons of receiving vaccination between the age groups. Based on our result, 20 to 64-year-old individual with higher educational attainment and being “*Managed by school or company*” was associated with higher influenza vaccination reception. We consider that spreading knowledge through education and management may have potential on increasing the vaccine coverage. For respondents aged ≥65 year-old, there was no association between education and influenza vaccine coverage. The major reason for not receiving vaccination was “No particular reason.”

### 4.1 Limitations and strengths

This study has several limitations. First, the research was an internet self-reported survey and has a probability of recall bias. Second, the proportions of older individuals were relatively small compared to participants of 20–64 years. Highly educated older respondents may have accessed the survey and the possibility of selection bias leading to overestimation of receiving vaccination cannot be denied. Third, regional information is one of the important variables in SES. Although the study obtained information of all the Japanese 47 prefectures, information on whether the residual area was urban or rural could not be further obtained. Fourth, the precise targeted population could not be obtained by the internet research company, making it difficult for us to calculate the response rate.

Previous studies have only conducted individually on health care workers (Eaton et al., 2017; Ishikane et al., 2016), school children (Kawai et al., 2011), elders (Zhou et al., 2013; Charu et al., 2011) or pregnant women (Meeting of the Strategic Advisory Group of Experts on immunization, 2015; Regan et al., 2016; Yamada et al., 2015). Therefore, our study had advantage of including population with wider age range. Also, we assume that the number of disproportionate distribution of respondents in the prefectures may affect our study results. Finally, data information lacked on the background of individuals, for instance, parental background, cognitive ability and risk cognition. These have possibilities of affecting uptake of influenza vaccination. Further considerations are need in the future.

## 5 Conclusion

Younger participants with higher educational attainment and higher household income had significantly higher reception of influenza vaccination. Although older adults aged ≥65 years have possible subsidies for influenza vaccination, the vaccination coverage did not differ between those aged <65 years and ≥65 years.

## Declaration of Competing Interest

The authors declare that there is no conflict of interest and no financial disclosures.
